# Variable Smear Layer and Adhesive Application: The Pursuit of Clinical Relevance in Bond Strength Testing

**DOI:** 10.3390/ijms20215381

**Published:** 2019-10-29

**Authors:** Abu Faem Mohammad Almas Chowdhury, Rafiqul Islam, Arefin Alam, Mariko Matsumoto, Monica Yamauti, Ricardo Marins Carvalho, Hidehiko Sano

**Affiliations:** 1 Department of Restorative Dentistry, Graduate School of Dental Medicine, Hokkaido University, Kita 13, Nishi 7, Kita-ku, Sapporo 060-8586, Japan; rony12cdc@den.hokudai.ac.jp (R.I.); arefin@den.hokudai.ac.jp (A.A.); mmatsu@den.hokudai.ac.jp (M.M.); myamauti@den.hokudai.ac.jp (M.Y.); sano@den.hokudai.ac.jp (H.S.); 2 Department of Oral Biological and Medical Sciences, Division of Biomaterials, Faculty of Dentistry, University of British Columbia, 2199 Wesbrook Mall, Vancouver, BC V6T 1Z3, Canada; Rickmc@dentistry.ubc.ca

**Keywords:** adhesion, dental bonding, smear layer, dentin-bonding agents, self-etch adhesive, universal adhesive, application mode, double application, bond strength, transmission electron microscopy

## Abstract

The removal or modification of smear layers that cover the dentin is critical to allow the penetration of adhesive molecules and to ensure a strong bond between resin and dentin. Aiming to establish a model for clinically-relevant dentin-bond testing, we evaluated the effects of smear layers created by abrasives having similar coarseness (180-grit SiC paper; fine-grit diamond bur) and application modes (single application; double application) on the microtensile bond strengths (µTBS) of two currently available universal adhesives (G-Premio Bond; Scotchbond Universal Adhesive) and a two-step self-etch adhesive (Clearfil Megabond 2). Sixty extracted human third molars were used for the μTBS test. Data were analyzed by three-way ANOVA and Tukey’s test (*α* = 0.05). Fracture modes were determined using stereomicroscopy. An additional 24 third molars were prepared for observation of the resin–dentin interface by TEM and adhesive-smear layer interaction by SEM. μTBS was significantly affected by the adhesives and their application modes (*p* < 0.001), implying that the double application of universal adhesives should be recommended to improve their performance. The effect of smear layers was not significant (*p* > 0.05), indicating that 180-grit SiC papers could be used to prepare dentin as a substitute for fine-grit diamond burs for dentin-bond testing in laboratory settings.

## 1. Introduction

The composite nature and inherent wetness of dentin make it a difficult substrate to use for bonding [1]. Additionally, in most clinical situations, dentin remains covered with smear layers, which hinders the penetration of adhesives molecules [2]. Therefore, the removal of this layer by an acid-etching step prior to the application of the bonding resin (etch-and-rinse technique) or modification by a self-etching monomer (self-etch technique) is crucial for creating a hybrid layer to ensure a strong bond between the resin and dentin [3]. To secure ideal bonding conditions, the acid demineralized dentin should be kept moist to prevent the collapse of collagen fibrils; at the same time, dentin should not be too wet, as excessive moisture will prevent collagen fibrils’ resin impregnation [4,5].

Self-etch adhesive systems can ensure the optimal wetness of dentin required for efficient bonding [6]. In addition, these systems are less technique-sensitive, require shorter application times [7], and induce little or no post-operative sensitivity [8]. Moreover, their one-step version further simplifies the bonding procedures [9]. Recently, the latest and most versatile version of one-step systems, i.e., universal adhesive systems, have been gaining more attention from clinicians [10]. These systems are more convenient because they can be applied both in self-etch and etch-and-rinse modes, as well as for direct and indirect restorations [11]. Moreover, their clinical evaluations have provided satisfactory outcomes [12,13]. Nonetheless, universal adhesive systems have been left with the shortcomings of their predecessors, i.e., one-step systems [14], and therefore, their bonding performance with dentin needs to be evaluated using different variables which are known to modify the bond strength results in vitro [15].

Bond strength testing is a commonly employed method to determine the effectiveness of adhesives in the laboratory [16]. Since the early days of adhesive research, 600-grit silicon carbide papers (SiC) (average particle: size 29 µm) have been the most commonly employed abrasives for creating standardized smear layers across bond strength testing laboratories [17,18,19,20,21]. They are also frequently used by manufacturers before launching new adhesive systems. Consequently, clinicians are often compelled to analogize these results with clinical substrates which are noticeably different. For instance, smear layers prepared with regular-grit diamond burs (average particle size: 100 µm) are rougher, thicker, and more compact than those of 600-grit SiC [22], and consequently, can compromise dentin bonding, especially when self-etch adhesives are used [23,24]. Therefore, the assimilation of the bond strength results obtained from 600-grit SiC-prepared smear layers may not present the precise scenario to the clinician, and can affect material choices, and ultimately, the quality of the bonded resin restoration. To address such issues, previous reports have suggested using a coarser SiC, such as 180-grit (average particle size: 63 µm) [25,26,27,28,29,30], or 120-grit (approximately 125 µm) and 400-grit SiC (approximately 35 µm) [31] for dentin preparation.

Many authors have suggested that doubling the application duration of one-step self-etch adhesives can improve monomer infiltration, resulting in increased bond strength to 180-grit SiC-prepared dentin [26,27,28,29,30]. Enhancing the application duration can also increase their residual water removal contributing positively to their bonding performance [30]. However, it is not yet known whether this enhanced application mode is equally beneficial for bonding through smear layers created in clinical situations. For instance, fine-grit diamond burs have similar coarseness (average particle size 60 µm) to 180-grit SiC, though it is yet to be evaluated if dentin smear layers created with fine-grit diamond burs would benefit from enhancing adhesives application duration. This evaluation would be important in validating the clinical relevance of the 180-grit SiC-prepared dentin and for standardizing substrate preparation to simulate clinical conditions for dentin-bond testing in laboratory settings.

The goal of this research was to establish an in vitro model for a clinically-relevant dentin-bond test. The objective of this study, therefore, was to evaluate the effects of smear layers prepared with fine-grit diamond burs and 180-grit SiC on the µTBS of the two current universal adhesives and a two-step self-etch adhesive applied to human dentin in single and double application modes. The tested hypotheses were: (1) the adhesives, (2) their application modes, and (3) the dentin smear layer variability would not have significant effects on the resin-dentin bond strengths.

## 2. Results and Discussion

### 2.1. Microtensile Bond Strengths (µTBS)

No pre-test failure was observed in this study. Three-way ANOVA revealed significant effects of adhesives (*F* = 135.363, *p* < 0.001) and their application modes (*F* = 49.042, *p* < 0.001) on the µTBS, but the effect of the smear layer was not significant (*F* = 1.201, *p* = 0.278). Also, there was statistically significant three-way interaction between these variables (*F* = 4.648, *p* < 0.05).

Smear layers are combinations of partially denatured collagen, other organic materials, and several minerals, based on the underlying dentin surface [32]. The variety of smear layer characteristics has been reported to affect resin-dentin bond strengths [22,31,33]. Interestingly, in the current study, smear layers prepared from abrasives having similar coarseness, i.e., 180-grit SiC (P) and fine-grit diamond bur (B), did not have a significant effect on the resin-dentin bond strengths of the tested adhesives. Tukey’s post-hoc test also revealed that regardless of the adhesive and application mode, the µTBS values obtained from bur (B) or SiC-prepared (P) dentin were not statistically different (*p* > 0.05), except for those with the Scotchbond Universal Adhesive (SB) with double application (D). These observations support our hypothesis that dentin smear layer variability would not have significant effects on the resin-dentin bond strengths. The µTBS test results are summarized in Table 1.

Our µTBS results are in agreement with a previous study by Sattabanasuk et al. [31], who also reported similar bond strengths when comparing different abrasive methods with similar coarseness: P120-grit SiC (approximately 125 µm) vs. medium-grit diamond bur (100 µm), and P400-grit SiC (approximately 35 µm) vs. fine-grit diamond bur (30 µm).

Although most self-etch adhesive systems are comprised of the same components, they can differ profoundly regarding the proportional amount of these components [16,34]. Consequently, specific limitations related to the composition of the adhesive might be considered to justify their bonding effectiveness. Contrary to the two-step self-etch adhesive Clearfil Megabond 2 (MB), one-step universal adhesives G-Premio Bond (GP) and SB contain more water for the dissociation of acidic functional monomers to be effective in self-etch approaches. Moreover, they need to be sufficiently hydrophilic in order to properly bond with “wet” dentin, yet at the same time, to become as hydrophobic as possible once polymerized to prevent water sorption and hydrolysis over time. Too much remaining water can also contribute to phase separation and incomplete polymerization of these materials [11]. Therefore, doubling the application time might increase water removal, contributing to improved bonding outcomes for one-step universal adhesives. In the current study, all adhesives demonstrated higher bond strengths in double application modes, though significantly, only for GP and SB with SiC-prepared dentin (*p* < 0.05). Moreover, SB and MB showed significantly higher µTBS than GP at all combinations (*p* < 0.05; Table 1). These observations rejected the hypotheses that adhesives and different application modes would not have significant effects on the resin-dentin bond strengths. Previous studies also reported significant improvements in bond strength with double application [26,27,28,29,30,35,36].

All the adhesives tested in this study contain the acidic monomer 10-MDP (Table 2), which possesses a high chemical affinity for hydroxyapatite (HAp), can interact chemically within the adhesives’ recommended application time [37], and is known to form stable calcium salts by nano-layering [38].

GP is an intermediately strong universal adhesive [pH 1.5], launched with an additional option to use the self-etch approach for dentin bonding without waiting for solvent evaporation (Manufacturer’s instructions, Japanese brochure). If successful, this recent innovation could constitute a big leap towards achieving optimal user-friendliness in dentin bonding. Unfortunately, the results of a previous study, aimed particularly at this evaluation, demonstrated that GP’s no-waiting self-etch approach resulted in lower bond strengths, thinner hybrid layers, and extensive nanoleakage [10]. In the present study, we also observed lower bond strengths for GP with single applications (10 s) following the manufacturer’s instructions (Table 1). In contrast, double applications (20 s) of GP contributed to increased bond strengths, probably by enhancing the dissolution of smear layer (discussed later under scanning electron microscopy-SEM observation), increasing the amount of acidic monomers (10-MDP and 4-META, Table 2) in direct contact with dentin, counteracting the buffering action of HAp, allowing more chemical interactions with 10-MDP, improving residual water removal, and infiltration of the adhesive into the demineralized dentin. Previous studies have also suggested similar mechanisms for the improvement of bond strengths with the double application of self-etch adhesives [30,35].

Despite being ultra-mild in nature (pH 2.7), the SB in this study might have also benefitted from its active application method, which ensures a persistent collision of the reactants [39], and from its constituent Vitrebond Copolymer (Table 2), which promotes additional chemical bonding with HAp [40]. Therefore, the active double application of uncured SB resulted in significantly higher bond strength values with 180-grit SiC-prepared dentin (*p* < 0.05). These findings are supplementary to our previous report about the bond strengths of GP and SB with 180-grit SiC-prepared dentin [30].

SB might have also benefited from the solubility parameters of HEMA/ethanol combinations, which are known to significantly increase bond strength to dentin by modifying the final degree of expansion of the dried matrix [41]. Moreover, ethanol/water combinations evaporate readily. This might explain why, either in single or double applications, the bond strength of SB was significantly higher than that of GP. Regarding GP, acetone, being highly volatile, cannot form an azeotrope with water, and therefore fails to promote adequate water evaporation, leading to decreased polymerization [42]. Moreover, the absence of HEMA results in phase separation, leading to a weak interface and premature bond failure. The strong acidic nature of GP works well for self-etching, but its shorter application time and highly volatile solvent probably prevent it from becoming sufficiently hydrophobic after polymerization, leading to lower bond strengths when used in single application mode. Double application helps to overcome these drawbacks by increasing water removal and resin infiltration into the hybrid layer. This improves the interfacial mechanical properties, and results in significantly higher bond strengths [30].

In the current study, the bonding performance of MB was not affected by smear layer variability or application modes. Similar observations were reported when the bond strengths of Clearfil SE Bond were evaluated against different smear layers [31] and by increasing the application time and adhesive thickness [27]. Our previous study employing Clearfil Megabond 2 also demonstrated similar findings [30]. Clearfil SE Bond is the predecessor of the newly-marketed Clearfil Megabond 2. Though MB contains 10-MDP, contrary to SB, its application does not include active rubbing. Therefore, in the absence of an induced persistent collision of the reactants, its mild acidic nature (pH 2) was probably not enough to bring about significant improvements with double application. Moreover, its low water content as a two-step self-etch adhesive might have also contributed to its similar bond strengths, despite that having led to an increase in the application time. The higher bond strength values of MB might have also resulted from its new photo initiator, which improves its degree of conversion, leading to enhanced mechanical properties, lower levels of water sorption, and higher bond strengths [43].

### 2.2. Fracture Modes

The µTBS values of the adhesives tested in this study were further supplemented by their failure patterns (Table 1). For the ease of explaining, we combined the mixed failure (M), cohesive failure in dentin (CD), and cohesive failure in composite-resin (CC) into the non-adhesive failure category (NA) [10]. In our previous study [30], we demonstrated that the hardness of the adhesive layers of SB and MB was significantly higher than that of GP, in both single and double application modes. We also observed that the improved interfacial mechanical property of SB and MB resulted in significantly higher bond strengths and increased percentages of non-adhesive failure compared to GP. The failure patterns of the current study showed similar results. Both SB and MB showed a predominance of non-adhesive failure (≥ 55%), implying a stronger adhesive layer, leading to higher bond strengths. GP, being the weakest among the three, showed predominantly adhesive failures (≥75%). GP with bur-cut dentin failed only in the adhesive layers (100%).

### 2.3. Transmission Electron Microscopy (TEM) Observation

**Figure 1:**
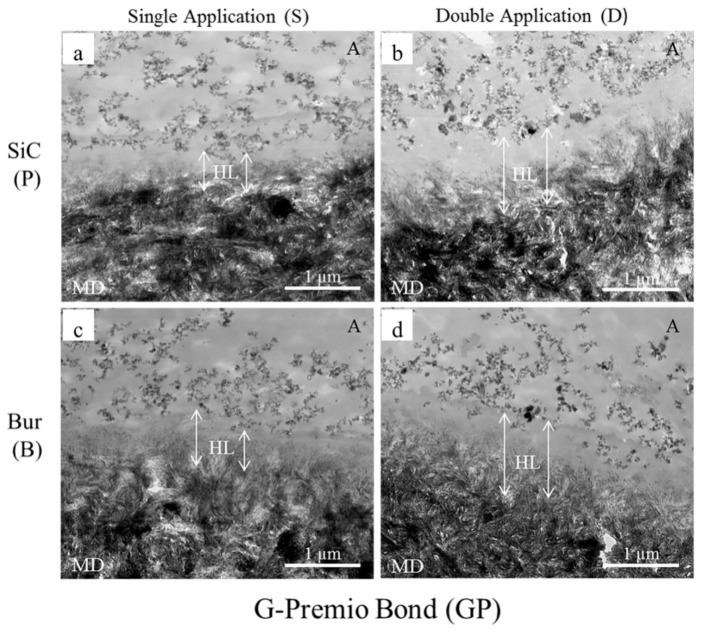
Representative TEM images (20,000×) of resin-dentin interfaces created by G-Premio Bond (GP) with 180-grit SiC paper-prepared (**a**,**b**) and fine-grit diamond bur-cut dentin (**c**,**d**) in single (**a**,**c**) and double application (**b**,**d**) modes. A represents an adhesive containing scattered nano-sized filler particles. The double-ended white arrows represent the extension of the hybrid layers (HL) from beneath the filler-containing adhesive layer until the electron-dense mineralized dentin (MD). The length of the arrows on either side of HL mark is different in bur-cut dentin, implying that the thicknesses are not uniform. The fillers could not get into the collagen webs of the HL, forming an electron-lucent, filler-free top part of the HL. Double applications (**b**,**d**) increased the thicknesses of the HL.

**Figure 2:**
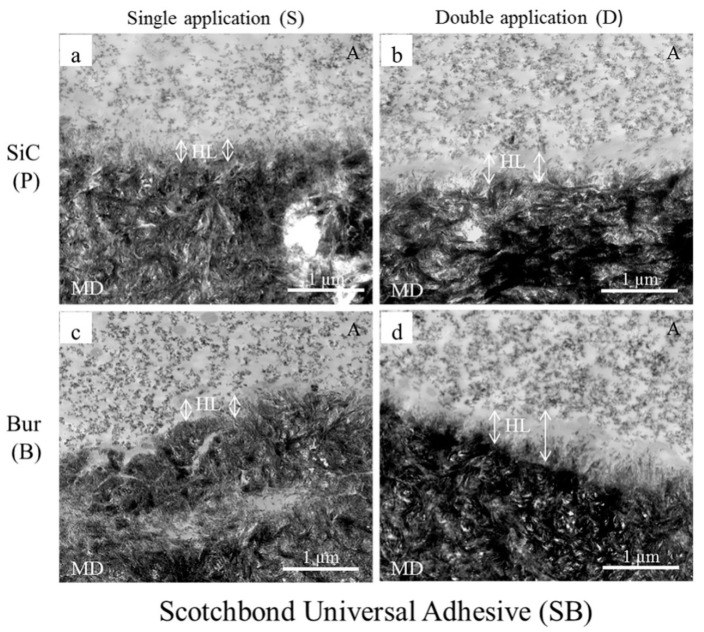
Representative TEM images (20,000×) of resin-dentin interfaces created by Scotchbond Universal Adhesive (SB) with 180-grit SiC paper-prepared (**a**,**b**) and fine-grit diamond bur-cut dentin (**c**,**d**) in single (**a**,**c**) and double application (**b**,**d**) modes. A represents an adhesive containing uniformly distributed nano-sized filler particles. In case of single applications (**a**,**c**), the morphologic features of interaction or demineralization is not as clear as was observed for G-Premio Bond (GP). The double-ended white arrows represent the extension of the hybrid layers (HL) from beneath the filler-containing adhesive layer until the electron-dense mineralized dentin (MD). The length of the arrows on either side of HL mark is different in bur-cut dentin, implying that the thicknesses are not uniform. Double applications (**b**,**d**) increased the thicknesses of the HL.

**Figure 3:**
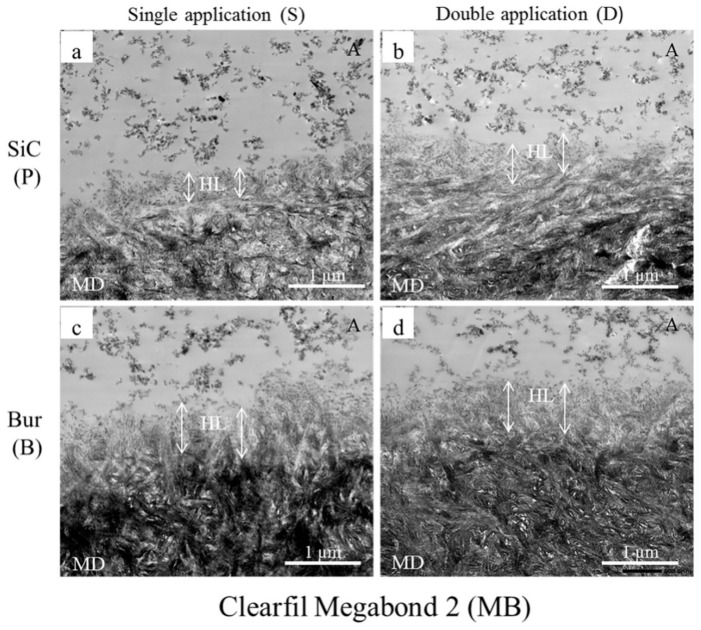
Representative TEM images (20,000×) of resin-dentin interfaces created by Clearfil Megabond 2 (MB) with 180-grit SiC paper-prepared (**a**,**b**) and fine-grit diamond bur-cut dentin (**c**,**d**) in single (**a**,**c**) and double application (**b**,**d**) modes. A represents an adhesive containing scattered nano-sized filler particles, albeit less scattered compared to what was observed in G-Premio Bond (GP). The double-ended white arrows represent the extension of the hybrid layers (HL) from beneath the filler-containing adhesive layer up to the electron-dense mineralized dentin (MD). Double application (**b**,**d**) did not yield marked changes from their single application counterparts (**a**,**c**), depicted in the images by similar lengths of the white arrows on either side of the HL.

Sattabanasuk et al. [31] demonstrated that SiC papers produced more irregular surfaces and thicker smear layers than their bur-prepared counterparts when the effects of abrasives with similar coarseness were compared. They also observed that SiC-prepared smear layers were associated with lower bond strength values in those situations. Our bond strength results also showed a similar trend, regardless of the application mode and adhesive, which were further complimented by the TEM images (Figure 1, Figure 2 and Figure 3).

Fillers are added to the adhesive to improve the strength of the adhesive layer, which, in turn, improves the bond strength [44]. Though adhesive layers (represented with A in the Figures) containing nano-sized filler particles could be seen in all our TEM images, the fillers were most concentrated and uniformly distributed in SB and least in GP. The fillers could not get into the underlying collagen webs of the hybrid layers (represented with HL in the Figures). These observations supplement the bond strength results of the tested adhesives.

The degree of filler loading is also associated with the rheological property of the adhesive [44], which would, in turn, determine the thickness and characteristic of the hybrid layer. Our TEM images showed that the thicknesses of the hybrid layers created by the adhesives/primer ranged from a few hundreds of nm to about 1500 nm. Generally, the thicknesses were more uniform with the 180-grit SiC-prepared smear layers than those of the fine-grit diamond bur-cut preparations, the latter being slightly thicker and wavy in appearance. Irrespective of the type of smear layer, double application (D) resulted in a noticeable increase of hybrid layer thicknesses in the cases of GP (Figure 1b,d) and SB (Figure 2b,d). In spite of having the shortest application time among the three tested adhesives, GP created the thickest hybrid layers in double application (D) modes (≥ 1000 nm), probably due to 10-MDP, and for the presence of the additional monomers 4-META and MDTP, which makes GP more acidic than the others (Table 2). The hybrid layers created with SB were thinner than those of GP, ranging from 200–300 nm in single application mode (S) to 600–700 nm in double application mode (D). The thicker hybrid layers of SB with double application (D) were probably the result of its active and enhanced application mode, as it contains 10-MDP [37,38,39]. In case of MB, despite containing 10-MDP, hybrid layers ranged between 500–600 nm in the SiC-prepared dentin to approximately 800–900 nm in bur-cut dentin, implying that double application (D) modes did not yield marked changes from their single application (S) counterparts (Figure 3). This observation could be explained by MB’s mild acidic nature, together with its inactive application. Overall, the formation of the thicker hybrid layers is probably related to the chemical and rheological characteristic of the adhesive/primer, i.e., increased acidity and less viscosity, as well as the application mode, i.e., enhanced application time and application type (active or not). The thicker hybrid layers observed from the TEM images of fine-grit diamond bur-prepared dentin probably indicated that the corresponding 180-grit SiC-prepared smear layers were thicker, which might have hindered resin penetration more than with the bur-prepared dentin. However, these observations are contrary to previous reports, where the effects of SiC and bur-prepared smear layers were compared without emphasizing the similarity of their coarseness [22,23].

### 2.4. Scanning Electron Microscopy (SEM) Observation

**Figure 4:**
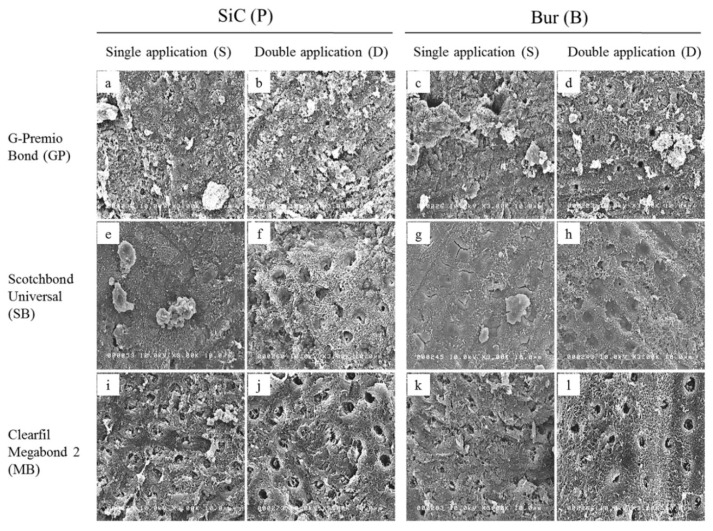
Representative SEM images (3000×) of 180-grit SiC paper-prepared (P) and fine-grit diamond bur-cut (B) dentin conditioned with the tested adhesives (GP and SB) and primer (MB) when applied in single (**a**,**c**,**e**,**g**,**i**,**k**) and double application mode (**b**,**d**,**f**,**h**,**j**,**l**).

Ground dentin surfaces treated with adhesives and primer were scanned to evaluate their characteristics and to relate to their bond strength values (Figure 4).

Remnants of the smear layer were more apparent in all single application (S) modes (Figure 4a,c,e,g,i,k), being most abundant in case of GP (Figure 4a,c), which may be related to its short application time; and least in case of SB (Figure 4e,g), which may be related to its active application. Double application (D) enhanced smear layer dissolution and exposure of the underlying dentin, including intertubular microporosities, dentinal tubules and HAp-depleted collagen fibrils, particularly in SB and MB (Figure 4f,h,j,l). Irrespective of the type of smear layer, there were still remnants of smear plugs within the dentinal tubules. GP and SB’s relative ineffectiveness against bur-cut dentin could be explained by their short application time and ultra-mild nature, respectively, as well as by the rougher and more compact nature of the bur-cut smear layers, making their chemical action insufficient to bring about significant improvements, even with double applications. This explanation is further substantiated by the less conspicuous smear layer dissolution in bur-cut double application modes compared to their SiC-prepared counterparts (Figure 4b vs. Figure 4d and Figure 4f vs. Figure 4h). The dentin surfaces depicted scratches and wavy appearances characteristic of SiC and bur grinding, respectively. All these observations are related to the application time and mode of the adhesives/primer. Nonetheless, for each adhesive, when corresponding application modes were compared, the overall characteristics of ground, treated dentin surfaces showed similar attributes, indicating the similarity of the morphological characteristics of the smear layers created with 180-grit SiC and fine-grit diamond bur.

In summary, the double application mode used in this study presents an easy technique that improved the quality of resin-dentin bonds, especially in case of universal adhesives. The similarity between the effects of different smear layers indicates that 180-grit SiC can be employed as a replacement of fine-grit diamond bur for substrate preparation in bond-testing laboratories.

## 3. Materials and Methods

### 3.1. Teeth Selection, Preparation, and Bonding Procedures

This study was conducted in accordance with the Declaration of Helsinki of 1975, revised in 2013, and the protocol was approved by the Research Ethics Committee of Hokkaido University Graduate School of Dental Medicine (Approval number 2013-07; Approval date 12 December 2013).

All teeth were stored in an aqueous solution of 0.5% Chloramine-T at 4 °C and used within 6 months of extraction. The adhesives and application methods used in this study are listed in Table 1. Sixty extracted sound human third molars were used for bond strength test [45]. The teeth were randomly allocated to 12 groups (*n* = 5) according to the adhesive systems: G-Premio Bond (GP; GC Corporation, Tokyo, Japan), Scotchbond Universal Adhesive (SB; 3M ESPE, Neuss, Germany) and Clearfil Megabond 2 (MB; Kuraray Noritake Dental Inc, Nigata, Japan); dentin smear layers: created either with 180-grit SiC papers (P; Sankyo-Rikagaku Co., Saitama, Japan) or fine-grit diamond burs (B; Diamond Point FG, F102R, ISO 223 090 016, Shofu Inc., Kyoto, Japan); and the adhesives application modes: single application according to manufacturers’ instructions (S) or double applications with light curing only after the application of the second coat (D).

Flat, occlusal dentin surfaces were exposed using a gypsum model trimmer under a water coolant, and subsequently checked with a light microscope to confirm that no enamel remained on the surface. Dentin surfaces were then further prepared (*n* = 30/abrasive) with either 180-grit SiC paper under running water for 60 s or fine-grit diamond point burs in a high-speed hand-piece with copious water spraying for 5 light pressure strokes per surface [22]. Each bur was discarded after the preparation of 5 surfaces. For single application (S) groups, adhesives were then applied according to the manufacturer’s instruction, and light-cured (Optilux 401, Demetron/Kerr, Orange, CA, USA) at ≥ 550 mW/cm^2^. For the double application (D) groups, light curing was done after the application of both coats. In the case of MB with double application (D), the application of two coats of primer was followed by the application of the bonding resin and light curing. After the application of the adhesives, approximately 4 mm thick layers of composite-resin (Clearfil AP-X, Kuraray Noritake Dental Inc., Okayama, Japan) were applied. The bonded teeth were then stored in distilled water at 37 °C for 24 h. Then, resin/dentin beams (cross-sectional area: 1 mm^2^) were prepared using a low-speed diamond saw (Isomet 1000, Buehler, Lake Bluff, IL, USA), according to the non-trimming technique. The four longest central beams were selected from each tooth and tested for µTBS.

### 3.2. µTBS Test

Each beam was attached to a Ciucchi’s jig with a cyanoacrylate adhesive (Model Repair II Blue, Dentsply-Sankin, Tokyo, Japan) and subjected to tensile force employing a 500-N load cell at a crosshead speed of 1 mm/min in a desktop testing apparatus (EZ-S, Shimadzu Co., Kyoto, Japan) until fracture occurred. Each beam was tested within 5 min after removal from water storage in order to prevent sample drying [46]. The tensile load causing fracture of each beam was recorded and divided with the cross-sectional area to achieve the µTBS in megaPascals (MPa). The mean bond strength of four beams derived from each tooth represented the µTBS value of that tooth, generating 5 µTBS values per group.

### 3.3. Fracture Mode Analysis

After the μTBS test, the two ends of the fractured specimens were examined with 10× magnification using a stereomicroscope. Fracture modes at the dentin sides of the specimens were taken into consideration and classified into the following categories: adhesive failure (A), cohesive failure in dentin (CD), cohesive failure in composite-resin (CC), and mixed failure (M).

### 3.4. TEM of Resin-Dentin Interface

An additional 12 third molars were prepared following the procedures described by Saikaew et al. [47] for TEM observation of the interface. Flat, occlusal dentin surfaces were exposed using a gypsum model trimmer, and the teeth were randomly allocated to 12 experimental groups. After preparation of the dentin surfaces with 180-grit SiC(P) or fine-grit diamond bur (B), adhesives were applied in specific application modes, as mentioned before, and light cured. After bonding, a very thin layer of unfilled resin (TEETHMATE F-1 2.0, Kuraray Noritake, Tokyo, Japan) was applied and light-cured. The bonded teeth were then stored in distilled water at 37 °C for 24 h. After water-storage, the bonded teeth were cut with a diamond saw to make approximately 2 mm thick dentin discs. Each disc was then cut into half perpendicularly to the adhesive/dentin interface. Each half was further cut into two rectangular sections perpendicular to the interface. The samples were then fixed overnight in 2.5% glutaraldehyde containing 0.1 M sodium cacodylate buffer at pH 7.4, which was followed by rinsing with the same buffer. Then, the specimens were dehydrated with a series of ascending grades of ethanol and embedded in epoxy resin (Epon 812, Polysciences, Inc, Warrington, PA, USA). A diamond knife (Diatome, Bienne, Switzerland) was then used in an ultramicrotome (Ultracut, UCT, Leica, Vienna, Austria) to obtain 60–90 nm thick sections through the resin-dentin interface. Finally, the sections were observed at 20,000× with a TEM (H-800, Hitachi, Tokyo, Japan) operating at 75 kV without staining.

### 3.5. SEM of Dentin Surfaces Treated with Adhesives and Primer

Flat, occlusal dentin surfaces of the additional 12 third molars were exposed with a diamond trimmer as mentioned previously. After the preparation of the dentin surfaces with 180-grit SiC (P) or fine-grit diamond bur (B), approximately 2.0 mm thick dentin discs were prepared using a diamond cut-off wheel. Each dentin disc was allocated to one of the twelve groups (mentioned before). After the application of the adhesive (GP and SB) or primer (MB) in the specific application modes, as mentioned before, and without light-curing, the discs were instantaneously immersed in 100% acetone for 1 min to remove the applied adhesives or primer [48], dehydrated with ethanol, and dried with hexamethyldisilazane [49]. The dentinal surfaces were then examined under SEM (3000×) to evaluate the morphological changes of the dentin [50].

### 3.6. Statistical Analysis

A parametric analysis of μTBS data was performed after confirming the normality (Shapiro-Wilk test) and homogeneity (Levene test). A three-way ANOVA was employed to demonstrate the effects of adhesives (i.e., GP, SB and MB), their application modes (i.e., S and D), dentin smear layers (i.e., P and B), and the interaction of these three factors on the µTBS results. Post-hoc multiple comparisons were done by a Tukey’s test at 5% level of significance. All statistical analyses were done by using SPSS 22.0 for Windows (SPSS, Chicago, IL, USA).

## 4. Conclusions

The results of this study demonstrated that dentin surface preparation with 180-grit SiC or fine-grit diamond bur did not affect the bond strength of the tested self-etch adhesives. This indicates that 180-grit SiC papers could also be employed to prepare clinically-relevant substrate conditions for dentin adhesion research in laboratory settings.

Moreover, the bonding performance of universal adhesives to dentin can be improved by doubling their application time.

## Figures and Tables

**Table 1:** Mean µTBS (MPa) ± standard deviations (SD) and percentage of fracture modes (A/CD/CC/M) *.

Adhesives	Smear Layer Created with	Single Application as Per Manufacturers’ Instruction (S)		Double Application with Curing after Second Coat (D)	
		µTBS ± SD	A/CD/CC/M	µTBS ± SD	A/CD/CC/M
G-Premio Bond (GP)	180-grit SiC (P)	25.7 ± 3.7 ^a^	95/5/0/0	39.8 ± 5.5 ^b^	75/0/0/25
	Fine diamond bur (B)	34.9 ± 4.4 ^a,b^	100/0/0/0	43.0 ± 2.9 ^b,c^	100/0/0/0
Scotchbond Universal Adhesive (SB)	180-grit SiC (P)	52.0 ± 4.3 ^c,d^	40/30/5/25	66.4 ± 4.5 ^e^	30/50/5/15
	Fine diamond bur (B)	51.7 ± 4.5 ^c,d^	30/40/0/30	53.0 ± 4.4 ^d^	30/20/0/50
Clearfil Megabond 2 (MB)	180-grit SiC (P)	56.1 ± 4.7 ^d^	20/60/5/15	59.3 ± 2.7 ^d,e^	35/55/0/10
	Fine diamond bur (B)	51.0 ± 4.4 ^c,d^	25/50/0/25	58.2 ± 6.3 ^d,e^	45/35/0/20

Different superscript lowercase letters indicate statistically significant differences between tested groups (Tukey’s test, *p* < 0.05). *A, adhesive failure; CD, cohesive failure in dentin; CC, cohesive failure in composite-resin; M, mixed failure. CD/CC/M, together, constitute the non-adhesive failure category [10].

**Table 2:** Adhesive system (batch number), composition, and application procedure.

Adhesives (Batch Number)	pH *	Composition	Single Application as Per Manufacturers’ Instruction (S)	Double Application with Curing after Second Coat (D)
Scotchbond Universal Adhesive (649958)	2.7	10-MDP, Vitrebond™ copolymer, HEMA, dimethacrylate resins, filler, silane, initiators, ethanol, water	1. Apply the adhesive and rub for 20 s. 2. Dry gently for about 5 s until it no longer moves and the solvent evaporates. 3. Light cure for 10 s.	1. Apply the adhesive and rub for 20 s. Repeat the step. 2. Dry gently for about 5 s until it no longer moves and the solvent evaporates. 3. Light cure for 10 s.
G-Premio Bond (1701111)	1.5	10-MDP, 4-META, MDTP, methacrylate acid ester, distilled water, acetone, photo initiators, fine powdered silica	1. Apply using a microbrush. 2. Leave undisturbed for 10 s. 3. Dry thoroughly with air under maximum air pressure. 4. Light cure for 10 s.	1. Apply using a microbrush. 2. Leave undisturbed for 10 s. 3. Repeat step 1 and 2. 4. Dry thoroughly with air under maximum air pressure. 5. Light cure for 10 s.
Clearfil Megabond 2 (000033)	2	Primer: 10-MDP, HEMA, hydrophilic aliphatic dimethacrylate, dl-CQ, water Bond: 10-MDP, Bis-GMA, HEMA, dl-CQ, hydrophobic aliphatic dimethacrylate, initiators, accelerators, silanated colloidal silica	1. Apply the primer and leave for 20 s. 2. Gentle air-blowing for > 5 s. 3. Apply the bond. 4. Gentle air-blowing to make the film uniform. 5. Light-cure for 10 s.	1. Apply the primer and leave for 20 s. 2. Repeat step 1. 3. Gentle air-blowing for >5 s. 4. Apply the bond. 5. Gentle air-blowing to make the film uniform. 6. Light-cure for 10 s.

10-MDP, 10-methacryloyloxydecyl dihydrogen phosphate; HEMA, 2-hydroxyethylmethacrylate; 4-META, 4-methacryloxyethyl trimellitic anhydride; MDTP, 10-methacryloxydecyl dihydrogen thiophosphate; CQ, camphorquinone; Bis-GMA, bisphenol-A-diglycidyl methacrylate. * Information as received from the manufacturers.

## Abbreviations

µTBS	Microtensile bond strength
SiC	Silicon carbide paper
SB	Scotchbond Universal Adhesive
A	Adhesive failure
CD	Cohesive failure in dentin
CC	Cohesive failure in composite-resin
M	Mixed failure
NA	Non-adhesive failure
MB	Clearfil Megabond 2
GP	G-Premio Bond
HAp	Hydroxyapatite
10-MDP	10-methacryloyloxydecyl dihydrogen phosphate
HEMA	2-hydroxyethylmethacrylate
4-META	4-methacryloxyethyl trimellitic anhydride
MDTP	10-methacryloxydecyl dihydrogen thiophosphate
CQ	Camphorquinone
Bis-GMA	Bisphenol-A-diglycidyl methacrylate
SEM	Scanning electron microscopy
TEM	Transmission electron microscopy
HL	Hybrid layer
MD	Mineralized dentin
